# 4-[(Benzyl­amino)­carbon­yl]-1-methyl­pyridinium bromide hemihydrate: X-ray diffraction study and Hirshfeld surface analysis

**DOI:** 10.1107/S2056989022003784

**Published:** 2022-04-12

**Authors:** Vitalii V. Rudiuk, Anna M. Shaposhnik, Vyacheslav M. Baumer, Igor A. Levandovskiy, Svitlana V. Shishkina

**Affiliations:** a Farmak JSC, 63 Kyrylivska str., Kyiv 04080, Ukraine; b Department of Organic, Chemistry, National Technical University of Ukraine, 37, Pobedy ave., Kyiv, 03056, Ukraine; c SSI "Institute for Single Crystals" NAS of Ukraine, 60 Nauky ave., Kharkiv, 61001, Ukraine; d V.N. Karazin Kharkiv National University, 4 Svobody sq., Kharkiv 61022, Ukraine

**Keywords:** 4-[(benzyl­amino)­carbon­yl]-1-methyl­pyridinium bromide, mol­ecular structure, crystal structure, Hirshfeld surface analysis

## Abstract

The hemihydrate of 4-[(benzyl­amino)­carbon­yl]-1-methyl­pyridinium bromide, C_14_H_15_N_2_O^+^·Br^−^·0.5H_2_O, was studied by single-crystal and powder X-ray diffraction methods. In the asymmetric unit, two organic cations of similar conformation, two bromide anions and one water mol­ecule are present. In the crystal, N—H⋯Br hydrogen bonds link the cations and anions. The formation of a set of inter­molecular C—H⋯Br and C—H⋯π inter­actions result in double chains extending parallel to [011]. A Hirshfeld surface analysis showed high contributions of H⋯H and C⋯H/H⋯C short contacts to the total Hirshfeld surfaces of the cations.

## Abstract 2

The ability of 4-[(benzyl­amino)­carbon­yl]-1-methyl­pyridinium bromide salt to form hydrates was studied. Hirshfeld surfaces analysis was performed for identification of inter­molecular inter­actions.

## 1. Chemical context

The 4-[(benzyl­amino)­carbon­yl]-1-methyl­pyridinium cation (Am^+^) has been shown to possess anti­viral activity (Buhtiarova *et al.*, 2003 ▸; Frolov *et al.*, 2004 ▸; Boltz *et al.*, 2018 ▸; te Velthuis *et al.*, 2021 ▸). Being charged due to quartenization of the pyridine N atom, this type of cation is more stable than its protonated analogue formed by H-atom transition in the form of an acid–base pair. Halogenide anions can be used as simple counter-ions of the organic cation. In fact, the iodide salt of 4-[(benzyl­amino)­carbon­yl]-1-methyl­pyridinium (AmI) is known as a multimodal anti­viral drug and has been studied by single-crystal X-ray diffraction, powder diffraction, IR spectroscopy, and DSC methods (Drebushchak *et al.*, 2017 ▸). The search for polymorphic modifications, hydrates or solvates is of great importance for the pharmaceutical industry to improve the quality of a drug and to protect intellectual property. However, polymorphic screening performed for the AmI salt did not reveal any other crystalline form. 
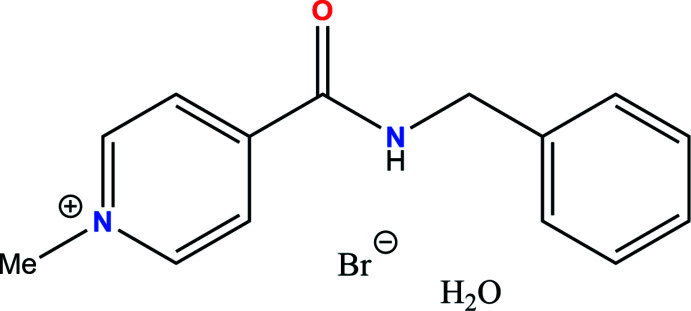


The 4-[(benzyl­amino)­carbon­yl]-1-methyl­pyridinium bro­mide (AmBr) salt is the closest analogue of AmI. Polymorphic screening for this salt resulted in the crystallization of a hemihydrate. In this communication we present the mol­ecular and crystal structures of 4-[(benzyl­amino)­carbon­yl]-1-methyl­pyridinium bromide hemihydrate, (C_14_H_15_N_2_O)^+^Br^−^·0.5H_2_O.

## 2. Structural commentary

**Figure 1:**
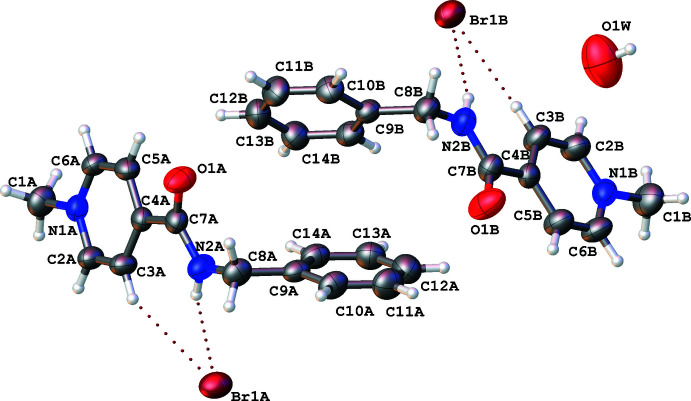
Mol­ecular structure of the title compound, AmBr hemihydrate. Displacement ellipsoids are shown at the 50% probability level. C—H⋯Br and N—H⋯Br hydrogen bonds are indicated by dotted lines.

**Figure 2:**
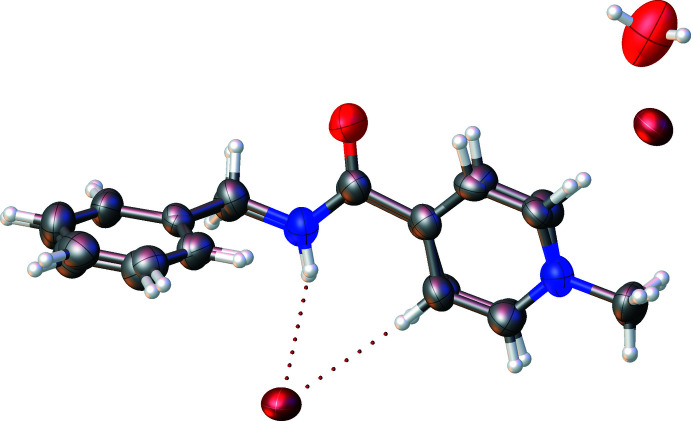
Mol­ecular overlay plot of cations *A* and *B*.

The asymmetric unit contains two mol­ecules of the cation (denoted *A* and *B*), two bromide anions (*A* and *B*) and one water mol­ecule (Fig. 1 ▸). The positive charge of the cation is located at the quaternized nitro­gen atom of the pyridine ring. The carbamide group is slightly non-coplanar with the plane of the aromatic ring, as shown by the N2—C7—C4—C3 torsion angles given in Table 1 ▸. The non-planarity is caused by steric repulsion between the two constituents as revealed by the _amide_H2⋯H3_pyridine_ and _amide_H2⋯C3_pyridine_ short contacts (Table 1 ▸) as compared to the van der Waals radii sums (Zefirov, 1997 ▸) of 2.34 and 2.87 Å, respectively. The cations *A* and *B* have similar conformations of the benzyl substituent (Fig. 2 ▸). The phenyl fragment of the benzyl substituent is located in an −*ac* position in relation to the C7—N2 bond and is twisted in relation to the carbamide fragment in both cations *A* and *B*, as seen in the C7—N2—C8—C9 and N2—C8—C9—C10 torsion angles (Table 1 ▸).

## 3. Supra­molecular features

**Figure 3:**
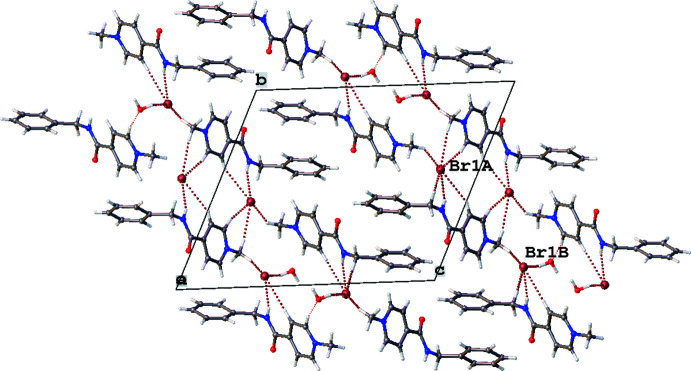
Crystal packing of AmBr hemihydrate in a view along [100]. Hydrogen-bonding inter­actions are shown by dashed lines.

In the crystal, cations *A* and *B* inter­act with the bromide anions by N—H⋯Br hydrogen bonds. In addition, a set of C—H⋯Br and C—H⋯π inter­actions are found in the crystal structure (Table 2 ▸). The solvent water mol­ecule forms one C—H⋯O hydrogen bond as a proton acceptor and O—H⋯Br and O—H⋯O hydrogen bonds as a proton donor (Table 2 ▸). All these hydrogen-bonding inter­actions result in the formation of double chains extending parallel to [011] (Fig. 3 ▸).

## 4. Hirshfeld surface analysis

**Figure 4:**
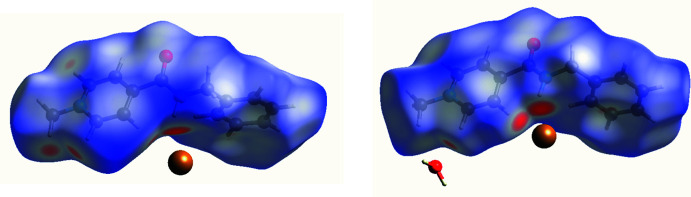
Hirshfeld surfaces mapped over *d*_norm_ for cations *A* (left) and *B* (right) in the crystal structure of AmBr hemihydrate.

**Figure 5:**
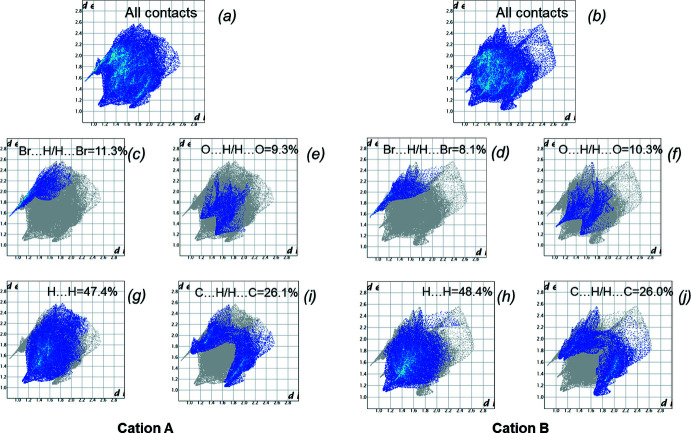
Contributions of inter­actions of different types to the total Hirshfeld surface of cations *A* and *B* in the crystal structure of AmBr hemihydrate.

Inter­molecular inter­actions were analysed using Hirshfeld surface analysis and two-dimensional fingerprint plots by using *CrystalExplorer17* (Turner *et al.*, 2017 ▸). The Hirshfeld surfaces were calculated separately for cations *A* and *B* using a standard high surface resolution, mapped over *d*_norm_ (Fig. 4 ▸). The red spots corresponding to contacts that are shorter than the van der Waals radii sum of the closest atoms are observed at the hydrogen atom of the amino group and at some phenyl and methyl hydrogen atoms. The two-dimensional fingerprint plots showed the absence of strong hydrogen bonds in the structure under study. To compare inter­molecular inter­actions of different types in a more qu­anti­tative way, their contributions to the total Hirshfeld surfaces were analysed (Fig. 5 ▸). The main contribution is provided by H⋯H short contacts (Fig. 5 ▸ *g*,*h*). The contribution of C⋯H/H⋯C short contacts is also significant (Fig. 5 ▸ *i*,*j*). The Br⋯H/H⋯Br and O⋯H/H⋯O inter­actions contribute to the total Hirshfeld surface in the same way (Fig. 5 ▸ *c*,*d* and 5*e*,*f*).

## 5. Database survey

A search of the Cambridge Structural Database (Version 5.42, update of November 2020; Groom *et al.*, 2016 ▸) revealed the structure of the anhydrous AmI salt with an equimolar cation:iodine ratio (refcode BEBFIA; Drebushchak *et al.*, 2017 ▸). A comparison of the mol­ecular conformation of the cation showed its flexibility due to rotation about the N—C*sp* ^3^ and C*sp* ^3^—C_ar­yl_ bonds.

## 6. Powder diffraction characterization

**Figure 6:**
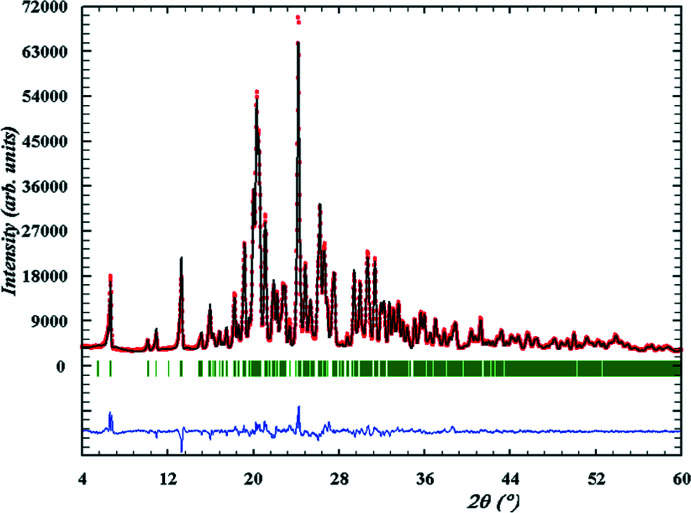
Final Rietveld plots for the title compound. Observed data points are indicated by red circles, the best-fit profile (black upper trace) and the difference pattern (blue lower trace) are shown as solid lines. The vertical green bars correspond to the Bragg reflections.

An X-ray powder diffraction pattern of the title compound was registered using a Siemens D500 powder diffractometer (Cu *K*α radiation, Bragg–Brentano geometry, curved graphite monochromator on the counter arm, 4 < 2*θ* < 60°, *D*2*θ* = 0.02°). A Rietveld refinement (Fig. 6 ▸) on the basis of the obtained pattern was carried out with *FullProf* and *WinPLOTR* (Rodriguez-Carvajal & Roisnel, 1998 ▸) using data of an external standard (NIST SRM1976) for the calculation of the instrumental profile function and the single-crystal data as the structure model for refinement. The main results of the Rietveld refinement are shown in Table 3 ▸. On the basis of the Rietveld refinement, the experimental powder X-ray diffraction pattern coincides with the theoretical one calculated from the X-ray single crystal study.

## 7. Synthesis and crystallization

4-[(Benzyl­amino)­carbon­yl]-1-methyl­pyridinium iodide (57.7 g, 0.163 mol), silver bromide (33.77 g, 0.180 mol) and 700 ml of water were loaded into a glass flask. The mixture was stirred for 72 h, and the resulting precipitate was filtered off. The solvent was evaporated under reduced pressure. To the precipitate were added 300 ml of aceto­nitrile and refluxed for 2 h. The reaction then was spontaneously cooled to a temperature of 303 K and the precipitate filtered off and rinsed on the filter with 50 ml of cooled aceto­nitrile. The product was dried at 313 K for 12 h. Yield: 14 g of 4-[(benz­yl­amino)­carbon­yl]-1-methyl­pyridinium bromide (28%); colourless crystals.

## 8. Refinement

Crystal data, data collection and structure refinement details are summarized in Table 4 ▸. All of the hydrogen atoms were placed in calculated positions and treated as riding with C—H = 0.96 Å, *U*_iso_(H) = 1.5*U*_eq_ for methyl groups and with C_ar_—H = 0.93 Å, C_*sp*_^2^—H = 0.97 Å, *U*_iso_(H) = 1.2*U*_eq_ for all other hydrogen atoms.

## Supplementary Material

Crystal structure: contains datablock(s) I. DOI: 10.1107/S2056989022003784/wm5623sup1.cif

Structure factors: contains datablock(s) I. DOI: 10.1107/S2056989022003784/wm5623Isup2.hkl

Supporting information file. DOI: 10.1107/S2056989022003784/wm5623Isup3.cml

CCDC reference: 2164796

Additional supporting information:  crystallographic information; 3D view; checkCIF report

## Figures and Tables

**Table 1:** Some geometrical characteristics (Å, °) of cations *A* and *B* in AmBr hemihydrate

Parameter	Cation *A*	Cation *B*
N1—C2	1.343 (6)	1.323 (7)
N1—C6	1.330 (7)	1.329 (7)
N2—C7—C4—C3	17.1 (7)	−1.4 (9)
C7—N2—C8—C9	−102.6 (6)	−107.0 (6)
N2—C8—C9—C10	−168.9 (5)	−167.4 (5)
H2⋯H3	2.11	2.04
H2⋯C3	2.59	2.54

**Table 2:** Hydrogen-bond geometry (Å, °) *CgA* and *CgB* are the centroids of the C9*A*–C14*A* and C9*B*–C14*B* rings, respectively.

*D*—H⋯*A*	*D*—H	H⋯*A*	*D*⋯*A*	*D*—H⋯*A*
N2*A*—H2*A*⋯Br1*A*	0.86	2.53	3.339 (5)	158
C3*A*—H3*A*⋯Br1*A*	0.93	2.98	3.814 (5)	150
C2*A*—H2*AA*⋯Br1*A* ^i^	0.93	2.84	3.725 (6)	159
C1*A*—H1*AA*⋯Br1*A* ^i^	0.96	2.88	3.784 (6)	157
C6*A*—H6*A*⋯*CgB* ^ii^	0.93	2.65	3.510 (7)	154
N2*B*—H2*B*⋯Br1*B*	0.86	2.60	3.419 (5)	159
C3*B*—H3*B*⋯Br1*B*	0.93	2.83	3.753 (5)	175
C6*B*—H6*B*⋯*CgA* ^iii^	0.93	2.71	3.400 (7)	132
O1*W*—H1*WA*⋯Br1*B* ^iv^	0.85	3.03	3.473 (7)	115
C1*A*—H1*AC*⋯O1*W* ^v^	0.96	2.89	3.794 (10)	157

Symmetry codes: (i) 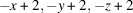 ; (ii) 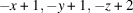 ; (iii) 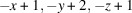 ; (iv) 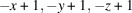 ; (v) 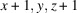 .

**Table 3:** Experimental data of the X-ray powder diffraction study performed at 293 K

Crystal system, space group	Triclinic, *P* 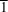
*a* (Å)	5.8858 (2)
*b* (Å)	14.7604 (3)
*c* (Å)	17.8118 (4)
*α* (°)	65.819 (1)
*β* (°)	85.321 (2)
*γ* (°)	85.402 (1)
*V* (Å^3^)	1405.09 (6)
*D*_*x*_ (Mg m^−3^)	1.499
Refinement	
*R*_p_	0.0359
*R*_wp_	0.0522
*R*_exp _	0.0120
*R*_B_	0.0371
*R*_F_	0.0171

**Table 4:** Experimental details

Crystal data	
Chemical formula	C_14_H_15_N_2_O^+^·Br^−^·0.5H_2_O
*M*_r_	316.19
Crystal system, space group	Triclinic, *P* 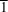
Temperature (K)	293
*a*, *b*, *c* (Å)	5.8891 (4), 14.7565 (10), 17.8090 (11)
α, β, γ (°)	65.773 (6), 85.396 (6), 85.544 (6)
*V* (Å^3^)	1405.08 (17)
*Z*	4
Radiation type	Mo *K*α
μ (mm^−1^)	2.92
Crystal size (mm)	0.30 × 0.15 × 0.10

Data collection	
Diffractometer	Xcalibur, Atlas
Absorption correction	Multi-scan (*CrysAlis PRO*; Rigaku OD, 2021 ▸)
*T*_min_, *T*_max_	0.634, 1.000
No. of measured, independent and observed [*I* > 2σ(*I*)] reflections	14465, 4925, 3547
*R*_int_	0.075
(sin θ/λ)_max_ (Å^−1^)	0.595

Refinement	
*R*[*F* ^2^ > 2σ(*F* ^2^)], *wR*(*F* ^2^), *S*	0.060, 0.175, 1.06
No. of reflections	4925
No. of parameters	339
H-atom treatment	H-atom parameters constrained
Δρ_max_, Δρ_min_ (e Å^−3^)	1.12, −0.45

Computer programs: *CrysAlis PRO* (Rigaku OD, 2021 ▸), *SHELXT* (Sheldrick, 2015*a* ▸), *SHELXL* (Sheldrick, 2015*b* ▸), *Mercury* (Macrae *et al.*, 2020 ▸), *OLEX2* (Dolomanov *et al.*, 2009 ▸).

## supplementary crystallographic information

### Crystal data

**Table:**

C_14_H_15_N_2_O^+^·Br^−^·0.5H_2_O	*Z* = 4
*M**_r_* = 316.19	*F*(000) = 644
Triclinic, *P*1	*D*_x_ = 1.495 Mg m^−^^3^
*a* = 5.8891 (4) Å	Mo *K*α radiation, λ = 0.71073 Å
*b* = 14.7565 (10) Å	Cell parameters from 4991 reflections
*c* = 17.8090 (11) Å	θ = 3.6–25.4°
α = 65.773 (6)°	µ = 2.92 mm^−^^1^
β = 85.396 (6)°	*T* = 293 K
γ = 85.544 (6)°	Plate, colorless
*V* = 1405.08 (17) Å^3^	0.30 × 0.15 × 0.10 mm

### Data collection

**Table:**

Xcalibur, Atlas diffractometer	4925 independent reflections
Radiation source: Enhance (Mo) X-ray Source	3547 reflections with *I* > 2σ(*I*)
Detector resolution: 10.3779 pixels mm^-1^	*R*_int_ = 0.075
ω scans	θ_max_ = 25.0°, θ_min_ = 3.4°
Absorption correction: multi-scan (CrysAlisPro; Rigaku OD, 2021)	*h* = −6→6
*T*_min_ = 0.634, *T*_max_ = 1.000	*k* = −16→17
14465 measured reflections	*l* = −21→20

### Refinement

**Table:**

Refinement on *F*^2^	0 restraints
Least-squares matrix: full	Hydrogen site location: mixed
*R*[*F*^2^ > 2σ(*F*^2^)] = 0.060	H-atom parameters constrained
*wR*(*F*^2^) = 0.175	*w* = 1/[σ^2^(*F*_o_^2^) + (0.0802*P*)^2^ + 0.8154*P*] where *P* = (*F*_o_^2^ + 2*F*_c_^2^)/3
*S* = 1.06	(Δ/σ)_max_ = 0.001
4925 reflections	Δρ_max_ = 1.12 e Å^−^^3^
339 parameters	Δρ_min_ = −0.45 e Å^−^^3^

### Special details

**Table:**

Geometry. All esds (except the esd in the dihedral angle between two l.s. planes) are estimated using the full covariance matrix. The cell esds are taken into account individually in the estimation of esds in distances, angles and torsion angles; correlations between esds in cell parameters are only used when they are defined by crystal symmetry. An approximate (isotropic) treatment of cell esds is used for estimating esds involving l.s. planes.

### Fractional atomic coordinates and isotropic or equivalent isotropic displacement parameters (Å^2^)

**Table:**

	*x*	*y*	*z*	*U*_iso_*/*U*_eq_	
Br1A	0.68503 (10)	1.06475 (4)	0.84634 (4)	0.0614 (2)	
Br1B	0.37067 (11)	0.44764 (5)	0.67323 (4)	0.0621 (2)	
O1A	0.3136 (7)	0.6956 (3)	0.9887 (2)	0.0611 (11)	
N1A	0.9794 (7)	0.7007 (3)	1.1351 (2)	0.0438 (10)	
N1B	0.6285 (7)	0.7967 (3)	0.3685 (3)	0.0496 (11)	
N2A	0.3986 (7)	0.8566 (3)	0.9181 (3)	0.0493 (11)	
H2A	0.486736	0.902420	0.913909	0.059*	
O1B	−0.0865 (8)	0.8195 (3)	0.5244 (3)	0.0766 (14)	
N2B	0.0287 (8)	0.6602 (4)	0.5982 (3)	0.0518 (11)	
H2B	0.133971	0.614443	0.603968	0.062*	
C9B	−0.1252 (8)	0.6264 (4)	0.7400 (3)	0.0413 (12)	
C9A	0.2998 (8)	0.8874 (4)	0.7765 (3)	0.0432 (12)	
C2A	0.9195 (9)	0.7968 (4)	1.0910 (3)	0.0473 (13)	
H2AA	0.996826	0.846374	1.096125	0.057*	
C10B	−0.2902 (9)	0.5848 (4)	0.8037 (3)	0.0505 (14)	
H10B	−0.423087	0.563252	0.792737	0.061*	
C4A	0.6308 (8)	0.7478 (4)	1.0305 (3)	0.0406 (12)	
C6A	0.8719 (9)	0.6286 (4)	1.1285 (3)	0.0440 (12)	
H6A	0.915307	0.562491	1.159801	0.053*	
C7A	0.4317 (8)	0.7654 (4)	0.9765 (3)	0.0431 (12)	
C14A	0.5157 (9)	0.8564 (4)	0.7586 (3)	0.0484 (13)	
H14A	0.618478	0.829285	0.800112	0.058*	
C3A	0.7436 (9)	0.8223 (4)	1.0381 (3)	0.0471 (13)	
H3A	0.701404	0.888839	1.007835	0.057*	
C8A	0.2168 (9)	0.8807 (4)	0.8609 (3)	0.0516 (14)	
H8AA	0.141562	0.943815	0.855334	0.062*	
H8AB	0.104777	0.830227	0.883854	0.062*	
C4B	0.2677 (9)	0.7633 (4)	0.4813 (3)	0.0435 (12)	
C6B	0.4700 (10)	0.8688 (4)	0.3605 (4)	0.0576 (15)	
H6B	0.481705	0.930081	0.315903	0.069*	
C13A	0.5825 (10)	0.8647 (4)	0.6799 (4)	0.0572 (15)	
H13A	0.730428	0.845033	0.668565	0.069*	
C5A	0.6988 (9)	0.6501 (4)	1.0763 (3)	0.0456 (12)	
H5A	0.626632	0.598873	1.071699	0.055*	
C8B	−0.1697 (9)	0.6367 (4)	0.6549 (3)	0.0517 (14)	
H8BA	−0.226852	0.574898	0.658879	0.062*	
H8BB	−0.287947	0.688592	0.632632	0.062*	
C14B	0.0695 (9)	0.6561 (4)	0.7590 (3)	0.0495 (13)	
H14B	0.182173	0.683315	0.717605	0.059*	
C10A	0.1499 (9)	0.9266 (4)	0.7139 (3)	0.0509 (14)	
H10A	0.003602	0.948655	0.724536	0.061*	
C13B	0.1036 (10)	0.6469 (4)	0.8380 (3)	0.0560 (14)	
H13B	0.236700	0.667865	0.849318	0.067*	
C11B	−0.2587 (10)	0.5752 (5)	0.8820 (4)	0.0600 (16)	
H11B	−0.370718	0.547599	0.923613	0.072*	
C1A	1.1677 (9)	0.6740 (5)	1.1928 (3)	0.0537 (15)	
H1AA	1.250770	0.732005	1.181669	0.080*	
H1AB	1.268614	0.623796	1.185187	0.080*	
H1AC	1.104851	0.649076	1.248552	0.080*	
C12B	−0.0633 (10)	0.6060 (5)	0.8998 (4)	0.0591 (15)	
H12B	−0.043285	0.599418	0.953119	0.071*	
C7B	0.0549 (9)	0.7494 (4)	0.5377 (3)	0.0490 (13)	
C2B	0.6158 (10)	0.7092 (4)	0.4317 (3)	0.0575 (15)	
H2BA	0.729127	0.659726	0.436849	0.069*	
C12A	0.4310 (11)	0.9020 (5)	0.6185 (4)	0.0653 (17)	
H12A	0.473938	0.905876	0.565843	0.078*	
C3B	0.4378 (10)	0.6904 (4)	0.4896 (3)	0.0540 (14)	
H3B	0.431425	0.628916	0.534200	0.065*	
C5B	0.2912 (10)	0.8543 (4)	0.4166 (3)	0.0560 (15)	
H5B	0.184373	0.906179	0.411098	0.067*	
C11A	0.2154 (11)	0.9334 (5)	0.6356 (4)	0.0658 (17)	
H11A	0.112389	0.959471	0.594095	0.079*	
C1B	0.8149 (11)	0.8123 (5)	0.3041 (4)	0.0697 (18)	
H1BA	0.752677	0.819184	0.253735	0.105*	
H1BB	0.922527	0.756296	0.321780	0.105*	
H1BC	0.890242	0.871600	0.295088	0.105*	
O1W	0.1099 (12)	0.5752 (7)	0.4262 (5)	0.132 (3)	
H1WA	0.222090	0.534776	0.446576	0.198*	
H1WB	0.054401	0.561287	0.389761	0.198*	

### Atomic displacement parameters (Å^2^)

**Table:**

	*U*^11^	*U*^22^	*U*^33^	*U*^12^	*U*^13^	*U*^23^
Br1A	0.0648 (4)	0.0476 (4)	0.0676 (4)	−0.0142 (3)	−0.0086 (3)	−0.0165 (3)
Br1B	0.0719 (4)	0.0510 (4)	0.0559 (4)	−0.0091 (3)	−0.0022 (3)	−0.0133 (3)
O1A	0.068 (3)	0.049 (2)	0.061 (2)	−0.0199 (19)	−0.0167 (19)	−0.0110 (19)
N1A	0.050 (2)	0.047 (3)	0.033 (2)	−0.0052 (19)	−0.0020 (17)	−0.015 (2)
N1B	0.056 (3)	0.052 (3)	0.041 (2)	−0.016 (2)	0.0023 (19)	−0.018 (2)
N2A	0.054 (3)	0.045 (3)	0.045 (2)	−0.0116 (19)	−0.0074 (19)	−0.011 (2)
O1B	0.069 (3)	0.059 (3)	0.075 (3)	0.002 (2)	0.018 (2)	−0.005 (2)
N2B	0.058 (3)	0.052 (3)	0.040 (2)	−0.003 (2)	0.0033 (19)	−0.015 (2)
C9B	0.044 (3)	0.038 (3)	0.039 (3)	−0.007 (2)	−0.002 (2)	−0.011 (2)
C9A	0.046 (3)	0.033 (3)	0.046 (3)	−0.007 (2)	−0.008 (2)	−0.009 (2)
C2A	0.062 (3)	0.036 (3)	0.043 (3)	−0.008 (2)	−0.009 (2)	−0.012 (2)
C10B	0.043 (3)	0.054 (4)	0.053 (3)	−0.015 (2)	0.007 (2)	−0.020 (3)
C4A	0.049 (3)	0.041 (3)	0.032 (3)	−0.007 (2)	0.001 (2)	−0.015 (2)
C6A	0.053 (3)	0.033 (3)	0.039 (3)	−0.003 (2)	−0.004 (2)	−0.008 (2)
C7A	0.049 (3)	0.045 (3)	0.036 (3)	−0.009 (2)	0.002 (2)	−0.016 (2)
C14A	0.048 (3)	0.041 (3)	0.052 (3)	−0.002 (2)	−0.011 (2)	−0.013 (2)
C3A	0.057 (3)	0.034 (3)	0.048 (3)	−0.006 (2)	−0.007 (2)	−0.013 (2)
C8A	0.045 (3)	0.051 (4)	0.052 (3)	0.001 (2)	−0.011 (2)	−0.013 (3)
C4B	0.054 (3)	0.043 (3)	0.033 (3)	−0.005 (2)	−0.005 (2)	−0.015 (2)
C6B	0.064 (4)	0.040 (3)	0.054 (3)	−0.007 (3)	0.003 (3)	−0.005 (3)
C13A	0.058 (3)	0.051 (4)	0.062 (4)	0.000 (3)	−0.004 (3)	−0.023 (3)
C5A	0.057 (3)	0.037 (3)	0.043 (3)	−0.012 (2)	0.003 (2)	−0.016 (2)
C8B	0.058 (3)	0.049 (3)	0.043 (3)	−0.009 (2)	0.000 (2)	−0.012 (3)
C14B	0.050 (3)	0.043 (3)	0.048 (3)	−0.010 (2)	0.005 (2)	−0.012 (3)
C10A	0.043 (3)	0.050 (3)	0.052 (3)	−0.001 (2)	−0.013 (2)	−0.011 (3)
C13B	0.060 (3)	0.058 (4)	0.055 (4)	−0.009 (3)	−0.007 (3)	−0.025 (3)
C11B	0.064 (4)	0.063 (4)	0.050 (3)	−0.019 (3)	0.018 (3)	−0.021 (3)
C1A	0.047 (3)	0.060 (4)	0.049 (3)	0.000 (3)	−0.014 (2)	−0.016 (3)
C12B	0.072 (4)	0.062 (4)	0.046 (3)	−0.004 (3)	−0.002 (3)	−0.025 (3)
C7B	0.051 (3)	0.051 (4)	0.039 (3)	−0.001 (3)	−0.003 (2)	−0.013 (3)
C2B	0.065 (4)	0.045 (4)	0.056 (4)	0.000 (3)	0.002 (3)	−0.015 (3)
C12A	0.079 (4)	0.061 (4)	0.061 (4)	−0.012 (3)	0.000 (3)	−0.029 (3)
C3B	0.063 (3)	0.045 (3)	0.042 (3)	−0.003 (3)	0.004 (2)	−0.007 (3)
C5B	0.058 (3)	0.045 (3)	0.054 (3)	0.001 (3)	0.004 (3)	−0.011 (3)
C11A	0.068 (4)	0.063 (4)	0.062 (4)	0.002 (3)	−0.022 (3)	−0.019 (3)
C1B	0.067 (4)	0.072 (5)	0.062 (4)	−0.012 (3)	0.019 (3)	−0.021 (3)
O1W	0.103 (5)	0.189 (8)	0.132 (6)	0.013 (5)	−0.021 (4)	−0.095 (6)

### Geometric parameters (Å, º)

**Table:**

O1A—C7A	1.223 (6)	C4B—C5B	1.373 (7)
N1A—C6A	1.330 (7)	C4B—C3B	1.382 (7)
N1A—C2A	1.343 (6)	C4B—C7B	1.514 (7)
N1A—C1A	1.491 (7)	C6B—C5B	1.357 (8)
N1B—C2B	1.323 (7)	C6B—H6B	0.9300
N1B—C6B	1.329 (7)	C13A—C12A	1.372 (9)
N1B—C1B	1.480 (7)	C13A—H13A	0.9300
N2A—C7A	1.332 (6)	C5A—H5A	0.9300
N2A—C8A	1.459 (7)	C8B—H8BA	0.9700
N2A—H2A	0.8600	C8B—H8BB	0.9700
O1B—C7B	1.231 (6)	C14B—C13B	1.386 (8)
N2B—C7B	1.326 (7)	C14B—H14B	0.9300
N2B—C8B	1.446 (7)	C10A—C11A	1.382 (8)
N2B—H2B	0.8600	C10A—H10A	0.9300
C9B—C14B	1.373 (7)	C13B—C12B	1.384 (8)
C9B—C10B	1.397 (7)	C13B—H13B	0.9300
C9B—C8B	1.502 (7)	C11B—C12B	1.375 (9)
C9A—C14A	1.376 (7)	C11B—H11B	0.9300
C9A—C10A	1.381 (7)	C1A—H1AA	0.9600
C9A—C8A	1.507 (8)	C1A—H1AB	0.9600
C2A—C3A	1.381 (8)	C1A—H1AC	0.9600
C2A—H2AA	0.9300	C12B—H12B	0.9300
C10B—C11B	1.370 (8)	C2B—C3B	1.370 (8)
C10B—H10B	0.9300	C2B—H2BA	0.9300
C4A—C5A	1.379 (7)	C12A—C11A	1.372 (9)
C4A—C3A	1.385 (7)	C12A—H12A	0.9300
C4A—C7A	1.515 (7)	C3B—H3B	0.9300
C6A—C5A	1.365 (8)	C5B—H5B	0.9300
C6A—H6A	0.9300	C11A—H11A	0.9300
C14A—C13A	1.383 (8)	C1B—H1BA	0.9600
C14A—H14A	0.9300	C1B—H1BB	0.9600
C3A—H3A	0.9300	C1B—H1BC	0.9600
C8A—H8AA	0.9700	O1W—H1WA	0.8506
C8A—H8AB	0.9700	O1W—H1WB	0.8502
			
C6A—N1A—C2A	120.9 (4)	C6A—C5A—H5A	120.0
C6A—N1A—C1A	119.3 (4)	C4A—C5A—H5A	120.0
C2A—N1A—C1A	119.9 (5)	N2B—C8B—C9B	114.0 (5)
C2B—N1B—C6B	120.5 (5)	N2B—C8B—H8BA	108.8
C2B—N1B—C1B	119.4 (5)	C9B—C8B—H8BA	108.8
C6B—N1B—C1B	120.0 (5)	N2B—C8B—H8BB	108.8
C7A—N2A—C8A	121.7 (5)	C9B—C8B—H8BB	108.8
C7A—N2A—H2A	119.1	H8BA—C8B—H8BB	107.6
C8A—N2A—H2A	119.1	C9B—C14B—C13B	122.1 (5)
C7B—N2B—C8B	122.7 (5)	C9B—C14B—H14B	119.0
C7B—N2B—H2B	118.6	C13B—C14B—H14B	119.0
C8B—N2B—H2B	118.6	C9A—C10A—C11A	120.6 (5)
C14B—C9B—C10B	117.7 (5)	C9A—C10A—H10A	119.7
C14B—C9B—C8B	123.6 (4)	C11A—C10A—H10A	119.7
C10B—C9B—C8B	118.7 (5)	C12B—C13B—C14B	119.1 (6)
C14A—C9A—C10A	118.3 (5)	C12B—C13B—H13B	120.5
C14A—C9A—C8A	123.7 (4)	C14B—C13B—H13B	120.5
C10A—C9A—C8A	118.0 (5)	C10B—C11B—C12B	120.7 (5)
N1A—C2A—C3A	120.4 (5)	C10B—C11B—H11B	119.7
N1A—C2A—H2AA	119.8	C12B—C11B—H11B	119.7
C3A—C2A—H2AA	119.8	N1A—C1A—H1AA	109.5
C11B—C10B—C9B	120.8 (5)	N1A—C1A—H1AB	109.5
C11B—C10B—H10B	119.6	H1AA—C1A—H1AB	109.5
C9B—C10B—H10B	119.6	N1A—C1A—H1AC	109.5
C5A—C4A—C3A	118.6 (5)	H1AA—C1A—H1AC	109.5
C5A—C4A—C7A	116.8 (5)	H1AB—C1A—H1AC	109.5
C3A—C4A—C7A	124.7 (5)	C11B—C12B—C13B	119.7 (6)
N1A—C6A—C5A	120.9 (5)	C11B—C12B—H12B	120.2
N1A—C6A—H6A	119.5	C13B—C12B—H12B	120.2
C5A—C6A—H6A	119.5	O1B—C7B—N2B	123.4 (5)
O1A—C7A—N2A	124.3 (5)	O1B—C7B—C4B	119.2 (5)
O1A—C7A—C4A	118.4 (5)	N2B—C7B—C4B	117.4 (5)
N2A—C7A—C4A	117.2 (5)	N1B—C2B—C3B	120.8 (5)
C9A—C14A—C13A	121.1 (5)	N1B—C2B—H2BA	119.6
C9A—C14A—H14A	119.4	C3B—C2B—H2BA	119.6
C13A—C14A—H14A	119.4	C13A—C12A—C11A	119.2 (6)
C2A—C3A—C4A	119.3 (5)	C13A—C12A—H12A	120.4
C2A—C3A—H3A	120.4	C11A—C12A—H12A	120.4
C4A—C3A—H3A	120.4	C2B—C3B—C4B	119.6 (5)
N2A—C8A—C9A	113.5 (4)	C2B—C3B—H3B	120.2
N2A—C8A—H8AA	108.9	C4B—C3B—H3B	120.2
C9A—C8A—H8AA	108.9	C6B—C5B—C4B	120.2 (5)
N2A—C8A—H8AB	108.9	C6B—C5B—H5B	119.9
C9A—C8A—H8AB	108.9	C4B—C5B—H5B	119.9
H8AA—C8A—H8AB	107.7	C12A—C11A—C10A	120.6 (5)
C5B—C4B—C3B	117.8 (5)	C12A—C11A—H11A	119.7
C5B—C4B—C7B	117.6 (5)	C10A—C11A—H11A	119.7
C3B—C4B—C7B	124.6 (5)	N1B—C1B—H1BA	109.5
N1B—C6B—C5B	121.0 (5)	N1B—C1B—H1BB	109.5
N1B—C6B—H6B	119.5	H1BA—C1B—H1BB	109.5
C5B—C6B—H6B	119.5	N1B—C1B—H1BC	109.5
C12A—C13A—C14A	120.2 (6)	H1BA—C1B—H1BC	109.5
C12A—C13A—H13A	119.9	H1BB—C1B—H1BC	109.5
C14A—C13A—H13A	119.9	H1WA—O1W—H1WB	109.4
C6A—C5A—C4A	120.0 (5)		
			
C6A—N1A—C2A—C3A	0.3 (8)	C14B—C9B—C8B—N2B	12.8 (8)
C1A—N1A—C2A—C3A	−179.3 (5)	C10B—C9B—C8B—N2B	−167.4 (5)
C14B—C9B—C10B—C11B	0.7 (8)	C10B—C9B—C14B—C13B	−0.7 (8)
C8B—C9B—C10B—C11B	−179.0 (6)	C8B—C9B—C14B—C13B	179.0 (5)
C2A—N1A—C6A—C5A	0.4 (8)	C14A—C9A—C10A—C11A	0.6 (8)
C1A—N1A—C6A—C5A	−180.0 (5)	C8A—C9A—C10A—C11A	−179.1 (6)
C8A—N2A—C7A—O1A	−1.5 (8)	C9B—C14B—C13B—C12B	0.3 (9)
C8A—N2A—C7A—C4A	177.9 (5)	C9B—C10B—C11B—C12B	−0.3 (10)
C5A—C4A—C7A—O1A	15.0 (7)	C10B—C11B—C12B—C13B	−0.1 (10)
C3A—C4A—C7A—O1A	−163.5 (5)	C14B—C13B—C12B—C11B	0.1 (9)
C5A—C4A—C7A—N2A	−164.4 (5)	C8B—N2B—C7B—O1B	−0.2 (9)
C3A—C4A—C7A—N2A	17.1 (7)	C8B—N2B—C7B—C4B	−178.4 (5)
C10A—C9A—C14A—C13A	0.5 (8)	C5B—C4B—C7B—O1B	−0.9 (8)
C8A—C9A—C14A—C13A	−179.8 (6)	C3B—C4B—C7B—O1B	−179.7 (6)
N1A—C2A—C3A—C4A	−0.5 (8)	C5B—C4B—C7B—N2B	177.4 (5)
C5A—C4A—C3A—C2A	−0.1 (8)	C3B—C4B—C7B—N2B	−1.4 (9)
C7A—C4A—C3A—C2A	178.4 (5)	C6B—N1B—C2B—C3B	0.9 (9)
C7A—N2A—C8A—C9A	−102.6 (6)	C1B—N1B—C2B—C3B	−176.0 (6)
C14A—C9A—C8A—N2A	11.4 (8)	C14A—C13A—C12A—C11A	1.9 (10)
C10A—C9A—C8A—N2A	−168.9 (5)	N1B—C2B—C3B—C4B	0.9 (10)
C2B—N1B—C6B—C5B	−0.3 (9)	C5B—C4B—C3B—C2B	−3.1 (9)
C1B—N1B—C6B—C5B	176.6 (6)	C7B—C4B—C3B—C2B	175.6 (6)
C9A—C14A—C13A—C12A	−1.8 (9)	N1B—C6B—C5B—C4B	−2.0 (10)
N1A—C6A—C5A—C4A	−1.0 (8)	C3B—C4B—C5B—C6B	3.7 (9)
C3A—C4A—C5A—C6A	0.8 (8)	C7B—C4B—C5B—C6B	−175.1 (6)
C7A—C4A—C5A—C6A	−177.8 (5)	C13A—C12A—C11A—C10A	−0.8 (10)
C7B—N2B—C8B—C9B	−107.0 (6)	C9A—C10A—C11A—C12A	−0.5 (10)

### Hydrogen-bond geometry (Å, º)

*CgA* and *CgB* are the centroids of the C9*A*–C14*A* and C9*B*–C14*B* rings, respectively.

**Table:**

*D*—H···*A*	*D*—H	H···*A*	*D*···*A*	*D*—H···*A*
N2*A*—H2*A*···Br1*A*	0.86	2.53	3.339 (5)	158
C3*A*—H3*A*···Br1*A*	0.93	2.98	3.814 (5)	150
C2*A*—H2*AA*···Br1*A*^i^	0.93	2.84	3.725 (6)	159
C1*A*—H1*AA*···Br1*A*^i^	0.96	2.88	3.784 (6)	157
C6*A*—H6*A*···CgB^ii^	0.93	2.65	3.510 (7)	154
N2*B*—H2*B*···Br1*B*	0.86	2.60	3.419 (5)	159
C3*B*—H3*B*···Br1*B*	0.93	2.83	3.753 (5)	175
C6*B*—H6*B*···CgA^iii^	0.93	2.71	3.400 (7)	132
O1*W*—H1*WA*···Br1*B*^iv^	0.85	3.03	3.473 (7)	115
C1*A*—H1*AC*···O1*W*^v^	0.96	2.89	3.794 (10)	157

Symmetry codes: (i) −*x*+2, −*y*+2, −*z*+2; (ii) −*x*+1, −*y*+1, −*z*+2; (iii) −*x*+1, −*y*+2, −*z*+1; (iv) −*x*+1, −*y*+1, −*z*+1; (v) *x*+1, *y*, *z*+1.
